# Engineering of *Bacillus thuringiensis* Cry2Ab toxin for improved insecticidal activity

**DOI:** 10.1186/s13568-024-01669-5

**Published:** 2024-02-01

**Authors:** Bai-Wen Fu, Lian Xu, Mei-Xia Zheng, Yan Shi, Yu-Jing Zhu

**Affiliations:** 1 Institute of Crop Sciences, Fujian Academy of Agricultural Sciences, Fuzhou, 350013 China; 2 School of Life Sciences, Xiamen University, Xiamen, 361005 China

**Keywords:** Cry2Ab, Protein engineering, Oligomerization, Pore-forming activity, Insecticidal activity

## Abstract

*Bacillus thuringiensis* Cry2Ab toxin was a widely used bioinsecticide to control lepidopteran pests all over the world. In the present study, engineering of *Bacillus thuringiensis* Cry2Ab toxin was performed for improved insecticidal activity using site-specific saturation mutation. Variants L183I were screened with lower LC_50_ (0.129 µg/cm^2^) against *P. xylostella* when compared to wild-type Cry2Ab (0.267 µg/cm^2^). To investigate the molecular mechanism behind the enhanced activity of variant L183I, the activation, oligomerization and pore-formation activities of L183I were evaluated, using wild-type Cry2Ab as a control. The results demonstrated that the proteolytic activation of L183I was the same as that of wild-type Cry2Ab. However, variant L183I displayed higher oligomerization and pore-formation activities, which was consistence with its increased insecticidal activity. The current study demonstrated that the insecticidal activity of Cry2Ab toxin could be assessed using oligomerization and pore-formation activities, and the screened variant L183I with improved activity might contribute to Cry2Ab toxin’s future application.

**Supplementary Information:**

The online version contains supplementary material available at 10.1186/s13568-024-01669-5.

## Introduction

*Bacillus thuringiensis* (Bt) is the most successful and widely used microbial insecticide to control agricultural pests such as lepidopterans, coleopterans, hemipterans and dipterans around the world. The pesticidal activities of Bt are mainly attributed to the δ-endotoxins generated during the sporulation stage, of which insecticidal crystal proteins (Cry toxins) are the most thoroughly studied δ-endotoxins (Aronson 2002; Bravo et al. 2007). Although the application of Cry toxins to Bt transgenic plants has been beneficial in controlling field pests, long-term and excessive usage of Cry toxins resulted in the resistance development of pests to Cry toxin. Up to now, more than ten species of pests have already been observed to develop resistance to Cry toxins in the field (Ferré & Van 2002). Among them, *Plutella xylostella* demonstrated remarkable resistance to Bt crops (Guo et al. 2015). *Helicoverpa armigera* in America and China evolved resistance to cotton expressing Cry1Ac toxin (Tabashnik et al. 1990, 2013) while *Spodoptera frugiperda* gained resistance to maize expressing Cry1Fa toxin (Storer et al. 2010). The development of pest resistance to Cry toxins significantly lowered the control efficacy of Cry toxins and jeopardized their long-term benefits, which became a key issue limiting their use.

Protein engineering is an important strategy for Cry resistance management because it can increase the stability of Cry toxin, improve its binding ability to pest midgut receptor, and enhance Cry toxin’s oligomerization or membrane perforation ability, all of which improve Cry toxin’s insecticidal activity and therefore help overcome insect resistance. (Soberón et al. 2007; Deist et al. 2014). To date, the majority of Cry toxins are made up of three domains, the first of which is commonly referred to as the pore formation domain. (Bravo et al. 2007; Vachon et al. 2002, 2012; Pardo-López et al. 2013). Researches on the critical residues of Cry2Ab toxin suggests that the helices α-4 and α-5 in Domain I are responsible for pore-formation, emphasizing the importance of this Domain in the process of Cry2Ab (Xu et al. 2018). Mutation at helix α-4 in the Domain I of Cry1A toxin can eliminate its toxicity to *Manduca sexta* larvae. However, these mutants exhibited no effect on the binding of Cry1A toxin to its midgut receptors, but they did have abnormalities in oligomerization and membrane insertion, resulting in the inability to generate pores on the cell membrane. (Girard et al. 2008). Replacing the residue Tyr202 into alanine or cysteine resulted in the full loss of Cry4A’s insecticidal action (Pornwiroon et al. 2004). It was also observed that site-directed mutagenesis improved Cry toxin’s ability to perforate the membrane. The elimination of the salt bridge might have improved the flexibility of Cry toxin, which could contribute to its perforating into the membrane. (Coux et al. 2001).

Our earlier studies found several critical residues involved in Cry2Ab oligomerization and insecticidal action (Pan et al. 2021). We predicted that engineering these residues would result in certain variants with increased insecticidal properties. As a result, site-specific saturation mutations were done at seven engineering locations, yielding the best variant L183I, with an LC50 that was 100% lower than wild-type Cry2Ab. The mechanism underlying improved activity in L183I was also investigated. Our findings identified a Cry2Ab mutant with increased activity, which could lead to its future application.

## Materials and methods

### Insects

*Plutella xylostella* larvae were purchased from Henan Jiyuan Baiyun Industry Co., Ltd, China. The artificial feed provided by Hubei Biopesticide Engineering Research Center was used to feed *P. xylostella* larvae under the conditions of temperature 26 to 27 ℃, humidity 30 to 40%, and photoperiod 14:10 h (light: dark).

### Preparation of *P. xylostella* midgut juice

*P. xylostella* migut justice (PxMJ) was prepared according to the method of Li et al. (2011). The *P. xylostella* at the end of the fourth instar was frozen on ice for 30 min before its midgut was dissected and extracted. The midgut was rinsed three times in a precooled sodium chloride solution (0.9%), then transferred to a glass homogenizer for fast ice bath homogenization. The homogenization solution was centrifuged at 4 ℃ at 15,000 rpm for 10 min. The supernatant is the *P. xylostella* migut juice. Protein concentration was quantifed with using a BCA Protein Assay Kit (biosharp, China) according to the manufacturer’s instructions.

### Site-specific saturation mutation

**Fig. 1:**
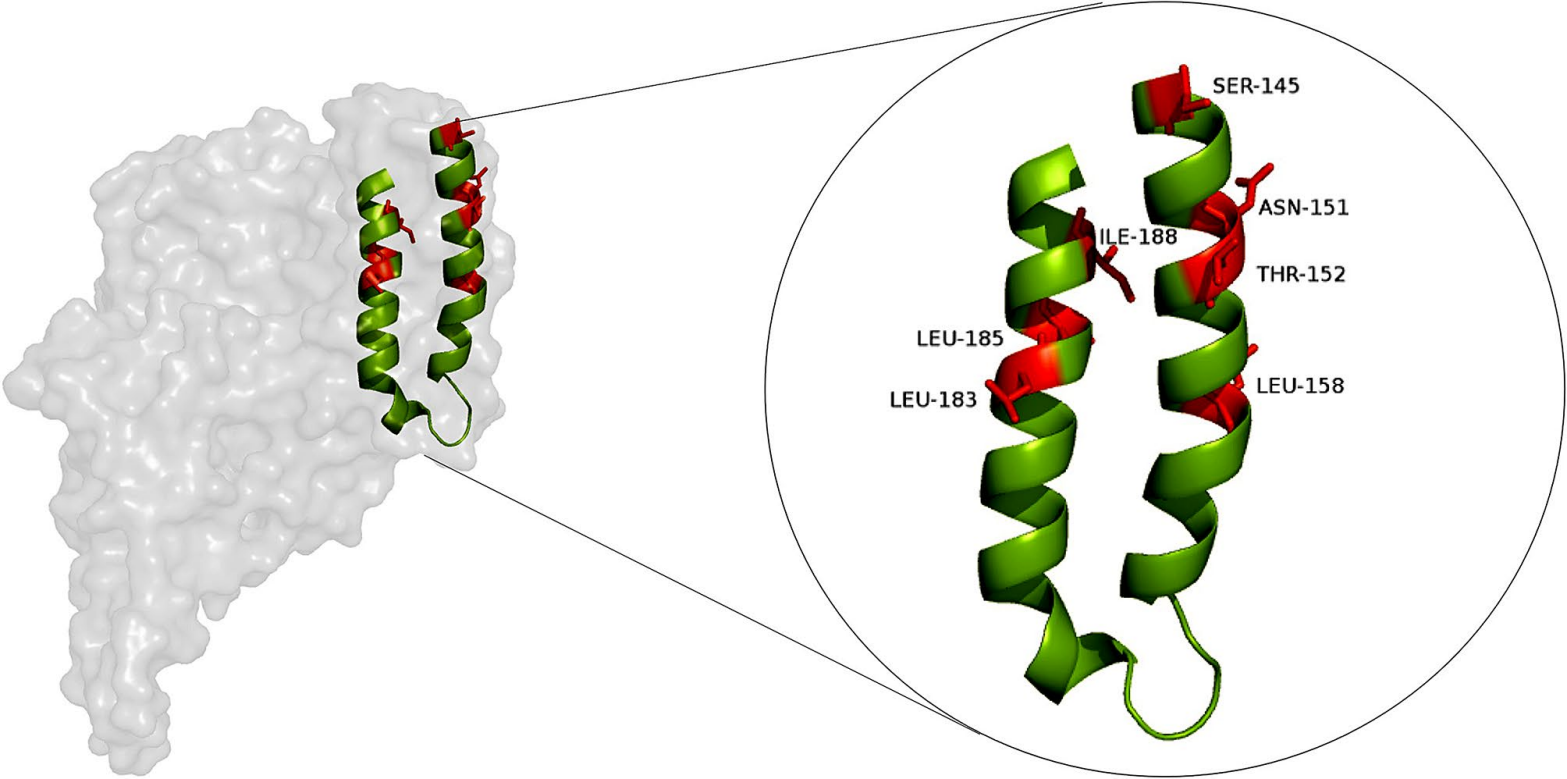
Key residues for engineering Cry2Ab for improved activity

Plasmid pET30-*cry2Ab* (NCBI accession number: EU623976) was constructed and kept in our lab (Pan et al. 2014). Seven potential engineering targets (S145, N151, T152, F157, L183, L185, I188) residing at α4-α5 in domain I of Cry2Ab (Fig. 1), were submitted for site-specific saturation mutation using plasmid pET30-*cry2Ab* as a template. Mutants were produced through polymerase chain reaction (PCR) using primers shown in Table S1. The PCR program was as follows: 98 °C for 2 min, (98 °C for 10 s, 55 °C for 10 s, 72 °C for 90 s) × 25 cycles, and a final elongation step at 72 °C for 5 min. After reaction, the PCR products (10 µL) were digested by *Dpn* I (0.1 µL) at 37 °C for 1 h, and directly transformed into competent *E. coli* BL21 (DE3) to create the relevant variant library.

### Expression and purification of Cry2Ab variants

The Cry2Ab variants were cultured in LB medium with 50 µg/ mL kanamycin. Cry2Ab variants were induced to expressed overnight at 25℃ with 0.1 mM isopropyl-β-D-thiogalactopyranoside (IPTG) after OD_600 nm_ reached 0.5. Cells were pelleted at 6000 g under 4℃ and were resuspended with Tris-HCl buffer (100 mM Tris-HCl, 200 mM NaCl, 25 mM imidazole, pH 7.0). Cells were lysed by ultrasonication and the soluble Cry2Ab protein was purified by a Ni-IDA Prepacked Column (Sangon, China) according to the instruction manual. The purified Cry2Ab mutant protein was displaced to a sodium carbonate buffer system (50 mM, pH 9.5) by a PD-10 desalting column (GE Healthcare, USA) before being analyzed by SDS-PAGE. Protein concentration was measured using a BCA Protein Assay Kit (Beyotime, China).

### Bioassay

The insecticidal toxicities of wild-type Cry2Ab and its mutants to *P. xylostella* were assessed by the method of Pan et al. (2013). Five or six concentrations of Cry2Ab toxins were set up, with sodium carbonate buffer (50 mM, pH 9.5) acting as a negative control. Each concentration assay employed twenty second instar larvae of *P. xylostella*, with three independent duplicates. Observations were recorded at 48 h, and the median lethal concentration (LC_50_) value was analyzed by SPSS 17.0 (Statistical Product and Service Solutions) using PROBIT analysis (Lewis and Finney 1972).

### Oligomerization assay

Wild-type Cry2Ab and its variants were activated by PxMJ and exchanged into sodium carbonate buffer (50 mM, pH 9.5) using a PD-10 desalting column.

For oligomeric formation assays, 20 µg of Cry2Ab activated-toxin was incubated at 30℃ ranging from 0 to 24 h in the present of PxBBMV (10 µg). Then the Cry2Ab toxins were mixed with 5×SDS-PAGE loading buffer (without 2-mercaptoethanol) and incubated at 60℃ for 10 min. The oligomeric formation of Cry2Ab was detected by 8% SDS-PAGE with coomassie staining. The ratio of oligomerization percentage was calculated by Image J software to evaluate the oligomerization proportion (oligomer / monomer × 100%).

### Liposome leakage assay

Liposome preparation was performed as described by Ding et al. (2016). Phosphatidylcholine, phosphatidylethanolamine and cholesterol were dissolved in chloroform and mixed in a 4:4:2 proportion (molar mass). The mixed lipids were put in a glass vial and evaporated under a stream of nitrogen to form lipid film. The lipid film was then shaken with SUV-1 buffer (20 mM HEPES, 50 mM NaCl, 3 mM calcein, pH 7.5) at room temperature for 2 h. Liposomes were formed by extruding the hydrated lipids through a 100-nm polycarbonate filter (Whatman) 35 times using a Mini-Extruder device (Avanti Polar Lipids Inc). Calcein outside the liposome was removed by exchanging the liposome with SUV-2 buffer (20 mM HEPES, 50 mM NaCl, pH 7.5) using a Sephadex G-50 column. Liposomes were stored at 4 °C and used within 48 h.

For liposome leakage assay, the liposome encapsulated calcein was diluted to 200 µM in SUV-2 buffer supplemented with 3 µM MnCl_2_. The released calcein could be quenched by MnCl_2_ in the solution. The excitation and emission wavelengths were set as 490 and 520 nm, respectively. Liposomes (480 µL) were introduced to the cuvette, and the emission fluorescence was measured as F_t0_. After adding 20 µL of activated-Cry2Ab (10 µg), the emission fluorescence (Ft) was continuously measured at 10-second intervals. After 10 min, 20 µL of 10% Triton X-100 was added to fully release the calcein and its fluorescence record was defined as F_t100_. The percentage of liposome leakage at each time point is defined as: leakage (t) (%) = (F_t_ - F_t0_) × 100 / (F_t100_ - F_t0_).

### Detection of root mean square deviation (RMSD) by molecular dynamic simulation

The structure model of wild-type Cry2Ab and variant L183I were constructed by Swiss-Model (https://swissmodel.expasy.org/) with the crystal structure of Cry2Aa protoxin structure (PDB: 1I5P) as a template (86.1% identity), and evaluated in SAVES (https://servicesn.mbi.ucla.edu/SAVES/).

The pretreatment of wild-type Cry2Ab and variant L183I for molecular dynamic (MD) were performed as previous study with AmberTools18. The system was heated from 0 to 303 K at constant volume in 50 ps with the protein restricted, equilibrated at constant pressure in 50 ps with the protein restricted and equilibrated for 500 ps without restriction of the protein. After equilibration, normal temperature and pressure (NPT) simulation was conducted for 8 ns to produce trajectories of MD simulation The root mean square deviation (RMSD) was calculated for the protein backbone atoms using least-square fitting derived from the MD trajectories (Xu et al. 2020).

## Results

### Engineering of Cry2Ab for improved insecticidal activity

Previous study identified key residues (S145, N151, T152, F157, L183, L185, I188) that were essential for Cry2Ab oligomerization and insecticidal activity (Fig. 1). In the present study, to improve the insecticidal activity of Cry2Ab, site-specific saturation mutation was accomplished on the seven residues listed above using degenerate codon NDT. Almost 200 individual colonies generated by site-specific saturation mutation were picked up in 24 deep-well plates containing 200 µL LB medium supplemented with 50 mg/L kanamycin. These colonies were submitted for DNA sequencing, yielding a total of 58 Cry2Ab mutants. These 58 Cry2Ab variants were further induced to expressed and purified. SDS-PAGE showed that the molecular weight of all the purified variants were about 65 kDa, which were consistent with the size of the wild-type Cry2Ab toxin (Fig S1).

The insecticidal activity of Cry2Ab mutants against the second instar of *P. xylostella* was tested, with wild-type Cry2Ab serving as a negative control. The LC_50_ of wild type Cry2Ab was 0.267 µg/cm^2^, as shown in Table 1. When compared to wild-type Cry2Ab, the insecticidal activities of most Cry2Ab variants were decreased. However, variants S145L, N151H and L183I, on the other hand, showed lower LC_50_ values. Among which L183I displayed the lowest LC_50_ (0.129 µg/cm^2^), highlighting its potent insecticidal properties. Besides, two variations, N151C and L185H, were rendered inactive.

**Table 1:** LC_50_ value of wild-type Cry2Ab and its variants against *P. xylostella* larvae

Cry2Ab	LC_50_ (µg/cm^2^)	95% confidence level (µg/cm^2^)	Relatively LC_50_	Slope ± SE^a^
Wild-type	0.267	0.135–0.487	100%	0.831 ± 0.041
S145C	0.666*	0.398–1.136	40.1%	0.858 ± 0.041
S145D	0.902*	0.436–2.065	29.6%	0.502 ± 0.036
S145F	0.398	0.276–0.567	67.1%	0.798 ± 0.040
S145G	0.278	0.176–0.427	96.0%	0.922 ± 0.043
S145H	0.678*	0.481–0.960	39.4%	0.965 ± 0.043
S145I	1.376*	0.801–2.588	19.4%	0.592 ± 0.037
S145L	0.206	0.126–0.322	129.6%	1.155 ± 0.051
S145N	0.299	0.189–0.458	89.3%	0.784 ± 0.04
S145R	0.314	0.189–0.503	85.0%	0.835 ± 0.041
S145V	0.425	0.297–0.606	62.8%	0.933 ± 0.043
S145Y	0.261	0.138–0.458	102.3%	0.775 ± 0.04
N151C	17.469*	no avail	1.5%	0.091 ± 0.033
N151D	0.653*	0.409–1.057	40.9%	1.023 ± 0.044
N151F	0.375	0.219–0.622	71.2%	0.797 ± 0.040
N151G	0.697*	0.447–1.098	38.3%	0.736 ± 0.039
N151H	0.167*	0.090–0.262	159.9%	0.778 ± 0.041
N151I	0.769*	0.491–1.225	34.7%	0.885 ± 0.041
N151L	0.476	0.290–0.777	56.1%	0.894 ± 0.042
N151R	0.482*	0.322–0.718	55.4%	0.743 ± 0.039
N151S	0.691*	0.461–1.041	38.6%	0.886 ± 0.041
N151V	0.214	0.121–0.355	124.8%	0.827 ± 0.042
N151Y	0.353	0.198–0.607	75.6%	0.671 ± 0.038
T152G	0.589*	0.315–1.275	45.3%	0.913 ± 0.173
T152H	0.351	0.199–0.639	76.1%	1.058 ± 0.181
T152I	0.537*	0.323–0.962	49.7%	1.190 ± 0.194
T152L	0.433	0.228–0.897	61.7%	0.900 ± 0.170
T152R	0.514*	0.287–1.011	51.9%	1.003 ± 0.177
T152S	0.486	0.255–1.038	54.9%	0.885 ± 0.171
T152V	0.442	0.257–0.806	60.4%	1.104 ± 0.185
F157G	0.313	0.127–0.783	85.3%	0.675 ± 0.160
F157H	0.386	0.204–0.764	69.2%	0.925 ± 0.173
F157L	0.407	0.191–0.959	65.6%	0.759 ± 0.164
F157N	0.372	0.217–0.670	71.8%	1.107 ± 0.185
F157Y	0.568*	0.270–1.454	47.0%	1.319 ± 0.201
L183C	0.478*	0.196–1.496	55.9%	1.183 ± 0.192
L183D	0.612*	0.305–1.461	43.6%	1.241 ± 0.198
L183F	0.451	0.285–0.743	59.2%	1.384 ± 0.209
L183G	0.543*	0.328–0.968	49.2%	1.211 ± 0.193
L183H	0.335	0.136–0.890	79.7%	1.119 ± 0.187
L183I	0.129*	0.052–0.278	207.0%	1.357 ± 0.220
L183N	1.175*	0.374–14.473	22.7%	1.572 ± 0.234
L183R	0.688*	0.299–2.116	38.8%	1.434 ± 0.217
L183S	0.485	0.307–0.806	55.1%	1.385 ± 0.211
L183V	0.489	0.287–0.888	54.6%	1.125 ± 0.187
L183Y	0.412	0.231–0.775	64.8%	1.025 ± 0.178
L185D	0.625*	0.309–1.519	42.7%	1.501 ± 0.228
L185G	0.365	0.190–0.731	73.2%	1.512 ± 0.226
L185H	>200*	no avail	<0.1%	0.222 ± 0.191
L185I	0.756*	0.326–2.526	35.3%	1.266 ± 0.209
L185R	0.560*	0.263–1.408	47.7%	1.444 ± 0.220
L185S	0.468	0.286–0.803	57.1%	1.249 ± 0.200
L185V	0.467	0.281–0.818	57.2%	1.198 ± 0.192
I188F	0.702*	0.385–1.511	38.0%	0.961 ± 0.176
I188G	0.638*	0.422–1.006	41.8%	1.651 ± 0.246
I188H	0.372	0.206–0.692	71.8%	1.013 ± 0.179
I188L	0.715*	0.423–1.339	37.3%	1.150 ± 0.194
I188R	0.380	0.205–0.735	70.3%	0.961 ± 0.175
I188S	0.409	0.214–0.835	65.3%	0.900 ± 0.170

*p < 0.05

### The mechanism for the improved insecticidal activity of L183I

**Fig. 2:**
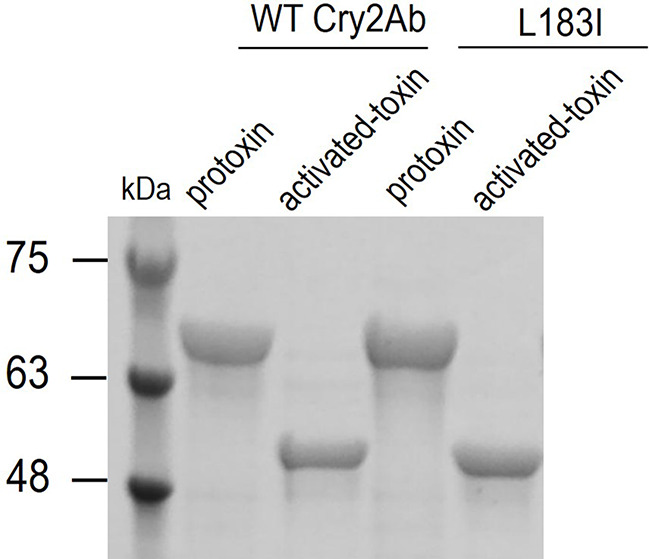
The proteolytic activation of wild-type Cry2Ab and variant L183I

The mode of action of Cry2Ab included three processes: activation by insect midgut protease, oligomerization and pore-formation. As a result, the activation, oligomerization, and pore-formation of L183I were examined with wild-type Cry2Ab as a control to investigate the mechanism behind the improved insecticidal action of L183I. The results in Fig. 2 reveal that both wild-type Cry2Ab and L183I could be cleaved into 50 kDa activated-toxin by *P. xylostella* midgut protease, implying that the increased insecticidal activity was not due to protoxin activation.

**Fig. 3:**
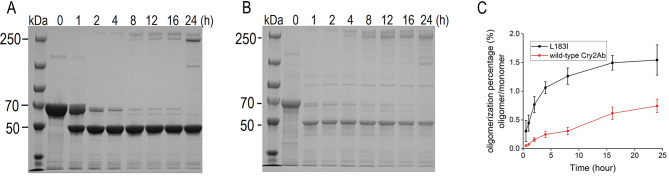
Oligomerization assay of wild-type Cry2Ab and variant L183I. **A** Time course of oligomerization of wild-type Cry2Ab. **B** Time course of oligomerization of L183I. **C** Oligomerization percentage of Cry2Ab. The data was calculated by image J and presented with the mean value ± standard deviations from triplicate biological experiments

The oligomerization of wild-type Cry2Ab and L183I were also evaluated (Fig. 3A and B). After 2 h, wild-type Cry2Ab could form 250 kDa oligomers, which was consistent with prior results (Xu et al. 2018). In L183I group, however, the oligomeric complex was found in 1 h, indicating that L183I’s oligomerization activity was stronger. We used Image J program to compute the proportion of Cry2Ab oligomer (oligomer / monomer 100%) to further calculate the oligomer formation of Cry2Ab. The proportion of oligomer and monomer in the L183I group was larger than in the wild-type Cry2Ab, as shown in Fig. 3C. The preceding results showed that L183I had better oligomerization activity than wild-type Cry2Ab, which could explain its improved insecticidal action.

**Fig. 4:**
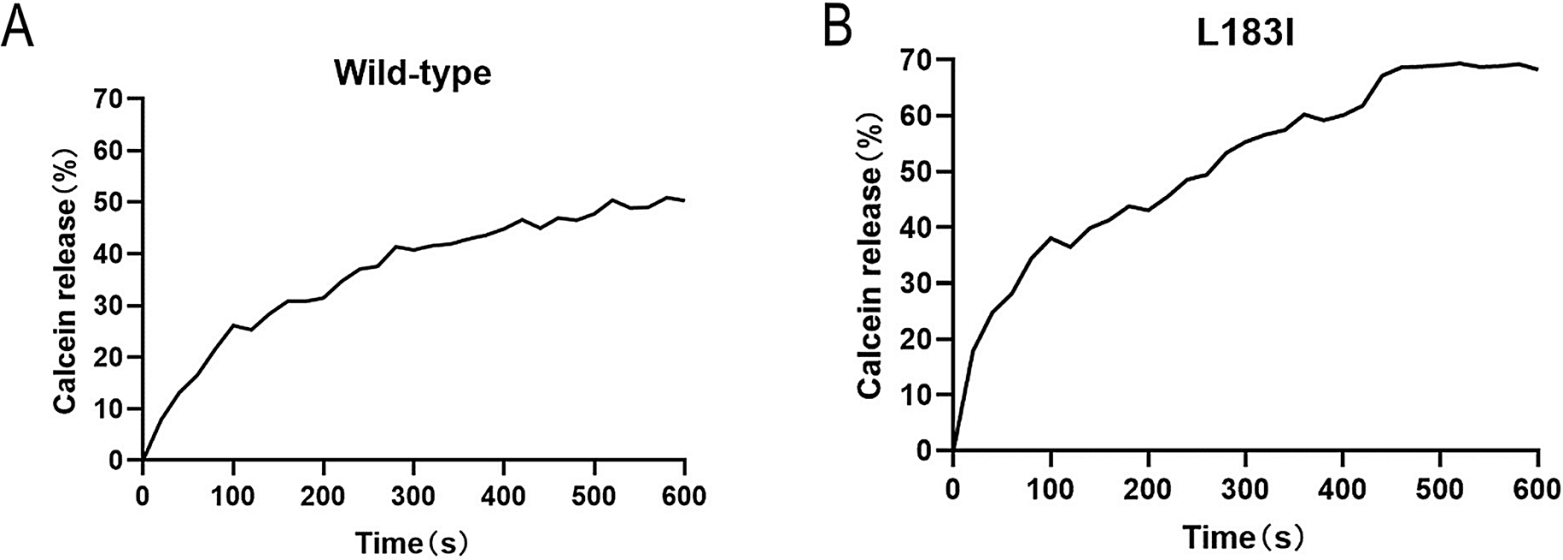
Liposome leakage assay of wild-type Cry2Ab and variant L183I. **A** Liposome leakage assay detected pore-forming activity of wild-type Cry2Ab. **B** Liposome leakage assay detected pore-forming activity of wild-type Cry2Ab

We then used the liposome leakage assay to evaluate the pore-forming activities of wild-type Cry2Ab and variant L183I. As seen in Fig. 4A and B. Both wild-type Cry2Ab and L183I could perforate liposomes, reducing the fluorescence intensity of the solution. Wild-type Cry2Ab, in particular, has been linked to 50.34% calcein leakage. In contrast, variation L183I increased the ability of liposome membrane penetration, resulting in 69.03% of calcein leakage, which is 40% more than wild-type Cry2Ab. Taken together, the results showed that Cry2Ab’s insecticidal action was connected to its oligomerization and pore-formation activities. Cry2Ab toxin is known for its ability to perforate the liposome membrane. Cry2Ab mutants that cannot oligomerize will have a reduced ability to perforate membranes. On the other hand, the mutants have a high degree of oligomerization as well as increased membrane perforation. It demonstrates that the degree of oligomerization is positively correlated with membrane piercing capabilities.

**Fig. 5:**
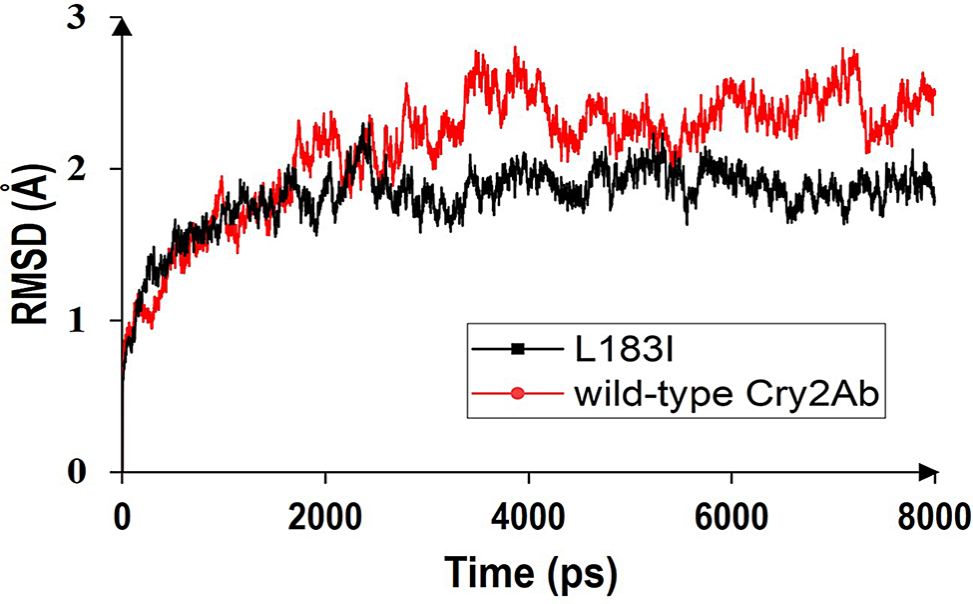
The RMSD values of wild-type Cry2Ab and variant L183I based on 8000 ps MD trajectories. The temperature was set as 303 K

To further understand the molecular mechanism behind the increased insecticidal effectiveness of variation L183I, the structures of wild-type Cry2Ab and L183I were build and submitted for 8 ns of molecular dynamics simulation. The root mean square deviation (RMSD) of wild-type Cry2Ab and L183I was calculated and compared as a result. The RMSD of wild-type Cry2Ab fluctuated more and had a higher mean value (2.15 Å) than L183I (1.91 Å), indicating that L183I were more stable in the overall structure. This could be the explanation for the better activity of L183I (Xu et al. 2020) (Fig. 5 and Table S2).

## Discussion

According to the reports by Girard et al. (2008) and Torres et al. (2008), domain I of Cry toxins plays a role in pore formation, specifically helices α-4 and α-5. It was reported that mutation on the Cry1Ab helix α-4 could resulted in the inactivation of Cry1Ab against *M. sexta* larvae. (Pacheco et al. 2020, 2023).

Likitvivatanavong et al. (2006) discovered that the residue N183 in Cry4Ba’s helices α-5 plays a role in oligomerization and membrane perforation. Xu et al. (2018) found that Cry2Ab activation requires proteolysis of helices α-4 and α-5. These results emphasized the significance of helices α-4 and α-5 in Domain I for the mode of action of Cry toxins.

Previous research revealed that the residues N151, T152, F157, L183, L185, and I188 were critical for Cry2Ab oligomerization and insecticidal action. As a result, we expected that modifying these residues might result in certain variants with improved activity. Saturation mutation was performed on the aforementioned residues, yielding a total of 58 mutants. The bioassay revealed that the majority of variations were negative; however, three variants, S145L, N151H, and L183I, had stronger insecticidal activity than wild-type Cry2Ab. L183I had the highest activity, with an LC50 of 0.129 µg/cm^2^.

Cry toxin’s pathogenicity is directly tied to its insecticidal action. The membrane perforation hypothesis is currently the commonly recognized perspective (Bravo et al. 2011, 2012; Alves et al. 2023). According to the hypothesis, prototoxin works in the midgut cavity of insect larvae and forms active monomeric toxin after trypsin activation in the midgut after larvae swallow cry prototoxin (Bravo et al. 2018). Cry monomeric toxin then contacts insect midgut receptors, causing Cry toxin to oligomerize. The oligomeric structure can interact with the lipid bilayer of the membrane to generate stable pores with a high opening probability, resulting in insect death (Schwartz et al. 1993; Rausell et al. 2004, Muñoz-Garay et al. 2006). Here we demonstrated the oligomerization and pore formation activity were closely related to insecticidal activity and pore-forming activity of Cry2Ab, since the variant L183I with improved activity showed higher oligomerization and pore-forming activity. Our results also suggested that engineering of Cry2Ab on Domain I was feasible for improved insecticidal activity. The molecular mechanism for the increased insecticidal activity of L183I is still unknown, so further study will focus on the structure basis of the replacement of leucine with isoleucine on the activity of Cry2Ab.

### Electronic supplementary material

Below is the link to the electronic supplementary material.

Supplementary Material 1

## Data Availability

The supplementary materials are available online.
