# Supplementary material for: Engineering of Bacillus thuringiensis Cry2Ab toxin for improved insecticidal activity
Source: AMB Express. 2024 Feb 1;14:15. doi: 10.1186/s13568-024-01669-5 (PMC10834393; doi:10.1186/s13568-024-01669-5)
Supplement: Supplementary file 1 — Supplementary Material 1 [file 13568_2024_1669_MOESM1_ESM.docx]

Supplementary material

**Engineering of *Bacillus thuringiensis* Cry2Ab toxin for improved insecticidal activity**

Bai-Wen Fu ^2^, Lian Xu ^1^, Mei-Xia Zheng ^1^, Yan Shi ^2^*, and Yu-Jing Zhu ^1^*

^1^ Agricultural Bio-Resources Research Institute, Fujian Academy of Agricultural Sciences, Fuzhou 350003, China.

^2^ School of Life Sciences, Xiamen University, Xiamen 361005, China.

* Corresponding authors:

Tel/Fax: 0591-83723032. E-mail: [zyjingfz@163.com](mailto:zyjingfz@163.com) (Yu-Jing Zhu)

Tel/Fax: 0592-2184648. E-mail: [yshi@xmu.edu.cn](mailto:yshi@xmu.edu.cn) (Yan Shi)

**Table S1.** **Primer sequence for producing mutant Cry2Ab through saturation mutation**

| primer | primer sequence (5’-3’) |
| --- | --- |
| Cry2Ab-NDT-145F | TCCTTTANDTATAACTTCTTCAGTTAATACAATGCAAC |
| Cry2Ab-AHN-145R | AAGTTATAHNTAAAGGAACAGCGTTTCGGTTAGG |
| Cry2Ab-NDT-151F | TTCAGTTNDTACAATGCAACAATTATTTCTAAATAGATTACCCCAG |
| Cry2Ab-AHN-151R | GCATTGTAHNAACTGAAGAAGTTATTGATAAAGGAACAGCG |
| Cry2Ab-NDT-152F | AGTTAATNDTATGCAACAATTATTTCTAAATAGATTACCCCAGTTCC |
| Cry2Ab-AHN-152R | GTTGCATAHNATTAACTGAAGAAGTTATTGATAAAGGAACAGCG |
| Cry2Ab-NDT-157F | ACAATTANDTCTAAATAGATTACCCCAGTTCCAGATGC |
| Cry2Ab-AHN-157R | TATTTAGAHNTAATTGTTGCATTGTATTAACTGAAGAAGTTATTGATAAAGG |
| Cry2Ab-NDT-183F | AGCCAATNDTCATCTTTCTTTTATTAGAGATGTTATTCTAAATGCAG |
| Cry2Ab-AHN-183R | AAAGATGAHNATTGGCTGCCTGTGCA |
| Cry2Ab-NDT-185F | TTTACATNDTTCTTTTATTAGAGATGTTATTCTAAATGCAGATGAATGGGG |
| Cry2Ab-AHN-185R | TAAAAGAAHNATGTAAATTGGCTGCCTGTGC |
| Cry2Ab-NDT-188F | TTCTTTTNDTAGAGATGTTATTCTAAATGCAGATGAATGGGG |
| Cry2Ab-AHN-188R | CATCTCTAHNAAAAGAAAGATGTAAATTGGCTGCCTG |

**Table S2.** DNA sequences of Cry2Ab.

| Cry2Ab, NCBI accession number: EU623976 |
| --- |
| DNA sequence:  ATGAATAGTGTATTGAATAGCGGAAGAACTACTATTTGTGATGCGTATAATGTAGCGGCTCATGATCCATTTAGTTTTCAACACAAATCATTAGATACCGTACAAAAGGAATGGACGGAGTGGAAAAAAAATAATCATAGTTTATACCTAGATCCTATTGTTGGAACTGTGGCTAGTTTTCTGTTAAAGAAAGTGGGGAGTCTTGTTGGAAAAAGGATACTAAGTGAGTTACGGAATTTAATATTTCCTAGTGGTAGTACAAATCTAATGCAAGATATTTTAAGAGAGACAGAAAAATTCCTGAATCAAAGACTTAATACAGACACTCTTGCCCGTGTAAATGCGGAATTGACAGGGCTGCAAGCAAATGTAGAAGAGTTTAATCGACAAGTAGATAATTTTTTGAACCCTAACCGAAACGCTGTTCCTTTATCAATAACTTCTTCAGTTAATACAATGCAACAATTATTTCTAAATAGATTACCCCAGTTCCAGATGCAAGGATACCAACTGTTATTATTACCTTTATTTGCACAGGCAGCCAATTTACATCTTTCTTTTATTAGAGATGTTATTCTAAATGCAGATGAATGGGGAATTTCAGCAGCAACATTACGTACGTATCGAGATTACTTGAAAAATTATACAAGAGATTACTCTAACTATTGTATAAATACGTATCAAAGTGCGTTTAAAGGTTTAAACACTCGTTTACACGATATGTTAGAATTTAGAACATATATGTTTTTAAATGTATTTGAGTATGTATCTATCTGGTCGTTGTTTAAATATCAAAGTCTTCTAGTATCTTCCGGTGCTAATTTATATGCAAGTGGTAGTGGACCACAGCAGACCCAATCATTTACTTCACAAGACTGGCCATTTTTATATTCTCTTTTCCAAGTTAATTCAAATTATGTGTTAAATGGATTTAGTGGTGCTAGGCTTTCTAATACCTTCCCTAATATAGTTGGTTTACCTGGTTCTACTACAACTCACGCATTGCTTGCTGCAAGGGTTAATTACAGTGGAGGAATTTCGTCTGGTGATATAGGTGCATCTCCGTTTAATCAAAATTTTAATTGTAGCACATTTCTCCCCCCATTGTTAACGCCATTTGTTAGGAGTTGGCTAGATTCAGGTTCAGATCGGGAGGGCGTTGCCACCGTTACAAATTGGCAAACAGAATCCTTTGAGACAACTTTAGGGTTAAGGAGTGGTGCTTTTACAGCTCGCGGTAATTCAAACTATTTCCCAGATTATTTTATTCGTAATATTTCTGGAGTTCCTTTAGTTGTTAGAAATGAAGATTTAAGAAGACCGTTACACTATAATGAAATAAGAAATATAGCAAGTCCTTCAGGAACACCTGGTGGAGCACGAGCTTATATGGTATCTGTGCATAACAGAAAAAATAATATCCATGCTGTTCATGAAAATGGTTCTATGATTCATTTAGCGCCAAATGACTATACAGGATTTACTATTTCGCCGATACATGCAACTCAAGTGAATAATCAAACACGAACATTTATTTCTGAAAAATTTGGAAATCAAGGTGATTCTTTAAGGTTTGAACAAAACAACACGACAGCTCGTTATACGCTTAGAGGGAATGGAAATAGTTACAATCTTTATTTAAGAGTTTCTTCAATAGGAAATTCCACTATTCGAGTTACTATAAACGGTAGGGTATATACTGCTACAAATGTTAATACTACTACAAATAACGATGGAGTTAATGATAATGGAGCTCGTTTTTCAGATATTAATATCGGTAATGTAGTAGCAAGTAGTAATTCTGATGTACCATTAGATATAAATGTAACATTAAACTCCGGTACTCAATTTGATCTTATGAATATTATGCTTGTACCAACTAATATTTCACCACTTTATTAA |

**Fig S1.**


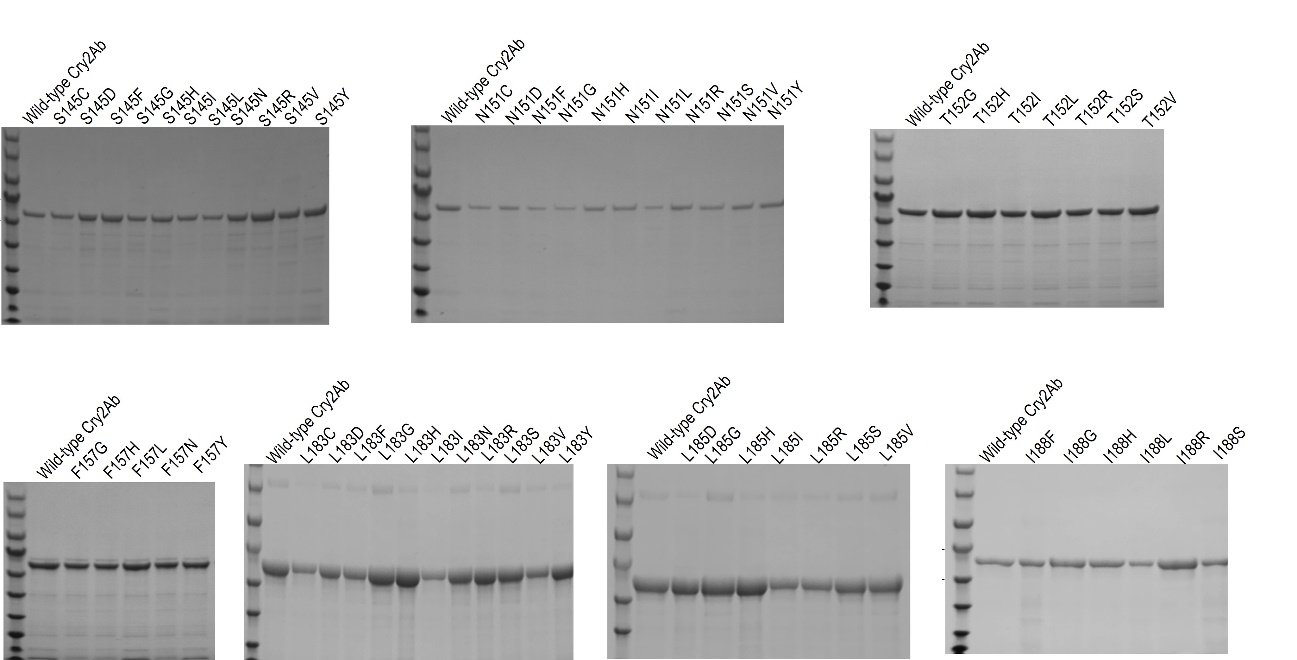


Fig S1. Purification of Cry2Ab variants.
